# Neoadjuvant Docetaxel/Cisplatin/5-Fluorouracil Enabling Laryngeal Preservation in Cervical Esophageal Carcinosarcoma: A Case Report

**DOI:** 10.70352/scrj.cr.26-0277

**Published:** 2026-07-07

**Authors:** Yohei Mizusawa, Tomoyoshi Kunitomo, Yasushige Takeda, Hijiri Matsumoto, Masashi Hashimoto, Naoaki Maeda, Shunsuke Tanabe, Yoshiyuki Ayada, Kazuhiro Noma

**Affiliations:** 1 Department of Gastroenterological Surgery, Okayama University Graduate School of Medicine, Dentistry and Pharmaceutical Sciences, Okayama, Okayama, Japan; 2 Department of Pathology and Oncology, Okayama University Graduate School of Medicine, Dentistry and Pharmaceutical Sciences, Okayama, Okayama, Japan

**Keywords:** esophageal neoplasms, carcinosarcoma, neoadjuvant therapy, docetaxel, cisplatin, fluorouracil, organ preservation, robotic surgical procedures

## Abstract

**INTRODUCTION:**

Esophageal carcinosarcoma is a rare malignancy comprising both epithelial and mesenchymal components, for which no standard treatment has been established. Organ preservation in cervical esophageal malignant tumors is particularly challenging because curative resection often necessitates laryngectomy. We describe a cervical esophageal carcinosarcoma that responded markedly to neoadjuvant docetaxel/cisplatin/5-fluorouracil (DCF), permitting laryngeal preservation, with a brief literature context.

**CASE PRESENTATION:**

A woman in her 50s presented with discomfort on swallowing. Upper endoscopy identified a type-1 polypoid tumor on the posterior wall 18 cm from the incisors, with involvement near the esophageal inlet at 17 cm. Biopsies showed a spindle-cell–predominant tumor; immunohistochemistry (cytokeratin AE1/AE3, p63) demonstrated an admixed epithelial component, supporting a diagnosis of esophageal carcinosarcoma. Contrast-enhanced CT revealed an approximately 6.5-cm exophytic lesion in the cervical to upper thoracic esophagus, and PET-CT showed intense uptake (maximum standardized uptake value 15.3). Clinical staging was cT3N0M0, cStage II (UICC TNM 8th edition). Two cycles of neoadjuvant DCF induced a dramatic response, leaving only a subtle ~5-mm proximal extension toward the right posterior wall. The patient underwent robot-assisted thoracoscopic subtotal esophagectomy with 3-field lymphadenectomy, followed by gastric conduit reconstruction through the posterior mediastinal route with cervical esophagogastric anastomosis using a 23-mm powered circular stapler. Intraoperative iodine staining delineated the proximal margin, and laryngeal preservation was achieved. Pathology showed pT1b-SM1, pN0, M0, pStage I with treatment-effect grade 1a, and the proximal resection margin was negative. Histology demonstrated a continuous transition between atypical squamous cells and spindle sarcomatous elements; the sarcomatous component exhibited inflammatory infiltrates predominantly composed of lymphocytes and foamy histiocytes, with focal hyalinization, consistent with a therapeutic effect. The postoperative course was uneventful, and the patient was discharged on day 16. No adjuvant therapy was administered. At 12 months of follow-up, no evidence of recurrence or metastasis has been observed.

**CONCLUSIONS:**

This rare case illustrates that neoadjuvant DCF can downstage cervical esophageal carcinosarcoma and enable curative, larynx-preserving resection. Such responses support consideration of neoadjuvant chemotherapy as a strategy for functional preservation in selected patients with this histology.

## Abbreviations

AE1/AE3
: anti-epithelial cytokeratin antibody AE1/AE3
DCF
: docetaxel/cisplatin/5-fluorouracil
HE
: hematoxylin and eosin
IHC
: immunohistochemistry
NBI
: narrow-band imaging
p63
: tumor suppressor protein p63
SUVmax
: maximum standardized uptake value

## INTRODUCTION

Esophageal carcinosarcoma is a biphasic malignant neoplasm composed of epithelial and mesenchymal components and accounts for 0.5%–2.8% of all esophageal malignancies.^1)^ Most tumors arise in the mid-to-lower esophagus, and occurrence in the cervical esophagus is even rarer.^2)^ No standard treatment strategy has been established; multidisciplinary management is generally modeled on squamous cell carcinoma, yet definitive evidence for the efficacy of chemotherapy—particularly against the sarcomatous component—remains limited. Reports of treatment achieving laryngeal preservation are also extremely scarce, highlighting the need to accumulate case-based evidence. We report a cervical esophageal carcinosarcoma in which neoadjuvant DCF achieved tumor shrinkage and enabled laryngeal preservation, together with clinical and pathological considerations.

## CASE PRESENTATION

**Fig. 1:**
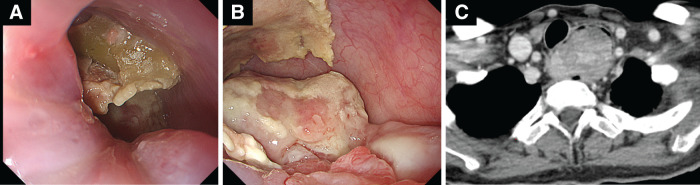
(**A**, **B**) Endoscopic images demonstrating a bulky type-1 protruding lesion on the posterior wall of the cervical esophagus, approximately 18 cm from the incisors. The tumor shows a polypoid configuration with an irregular surface covered by whitish exudate, suggestive of carcinosarcoma. (**C**) Contrast-enhanced CT showing an approximately 6.5-cm exophytic mass in the cervical esophagus without evidence of extramural invasion, lymph node enlargement, or distant metastasis.

**Fig. 2:**
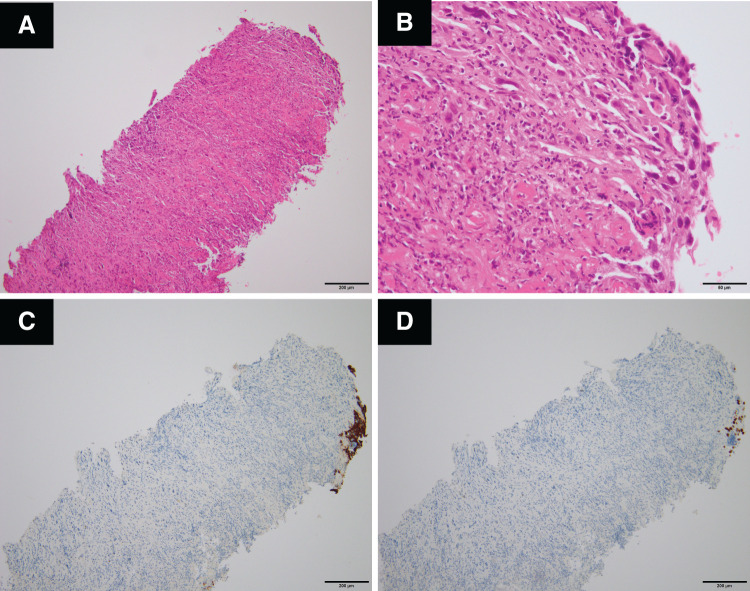
(**A**, **B**) Hematoxylin–eosin staining shows densely proliferating spindle-shaped and highly pleomorphic tumor cells, suggesting a predominantly mesenchymal component. (**C**) IHC for cytokeratin AE1/AE3 and (**D**) p63 demonstrate focal positivity in limited areas of the tumor, indicating coexistence of an epithelial (squamous cell carcinoma) component. The spindle-cell component is negative for both markers and is interpreted as a sarcomatoid element. IHC, immunohistochemistry

**Fig. 3:**
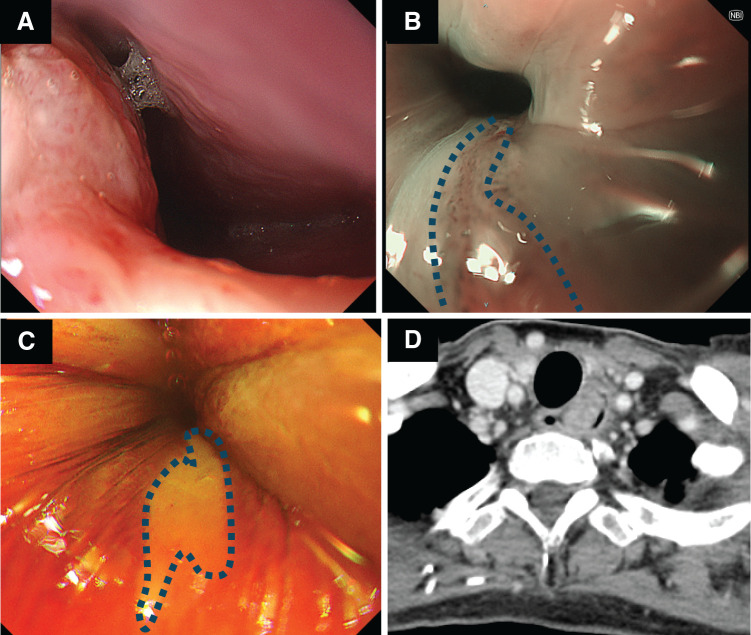
(**A**) Endoscopic examination demonstrating marked shrinkage of the protruding tumor in the cervical esophagus. (**B**) NBI showing a slightly irregular mucosal area on the posterior wall (dotted line). (**C**) Lugol’s iodine staining demonstrating a localized unstained area corresponding to the same region (dotted line), suggestive of residual disease. (**D**) Contrast-enhanced CT showing a significant reduction of the previously observed esophageal wall thickening. NBI, narrow-band imaging

**Fig. 4:**
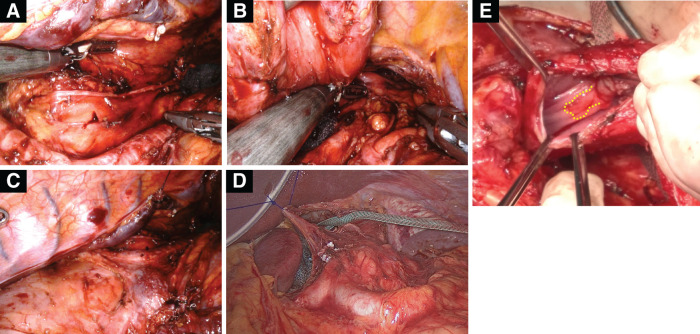
(**A**) Dissection of the left upper mediastinum. (**B**) Dissection of the right upper mediastinum. (**C**) Dissection of the middle mediastinum. (**D**) Abdominal lymph node dissection. (**E**) The proximal resection margin was confirmed by iodine staining to identify the tumor boundary, allowing laryngeal preservation.

**Fig. 5:**
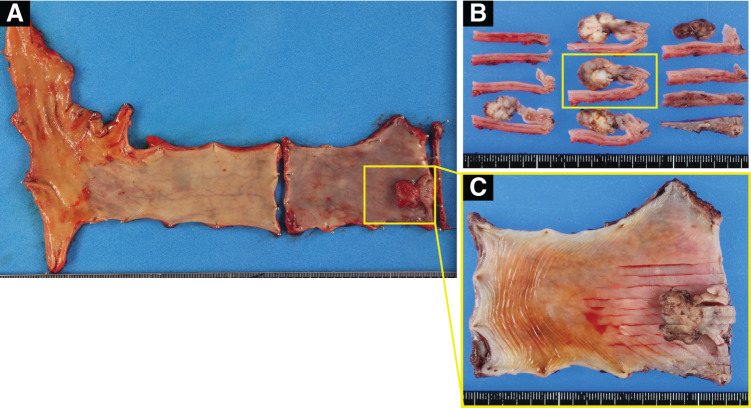
(**A**) The resected esophageal specimen showing a type-1 tumor mainly located on the posterior wall. (**B**) Serial sectioning of the specimen revealing a localized elevated tumor. (**C**) Mapping of the resected specimen. The yellow line indicates the area of submucosal invasion. Pathological findings: pT1b-SM1, pN0 (0/57), M0, Stage I (UICC TNM 8th Edition). The pathological treatment effect was graded as 1a according to the Japanese Classification of Esophageal Cancer.

**Fig. 6:**
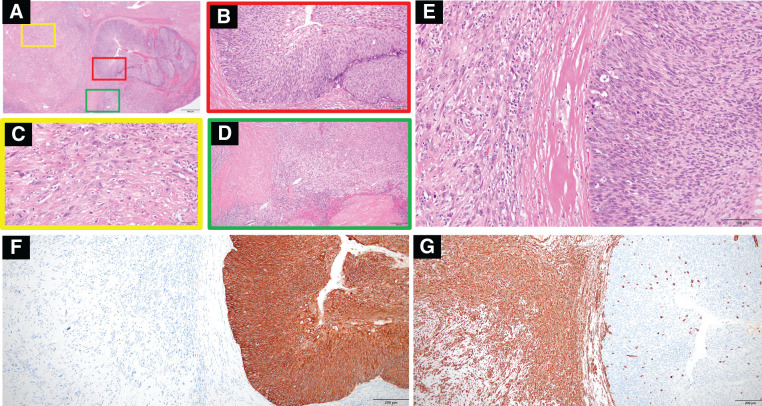
(**A**) Low-power hematoxylin–eosin staining demonstrating biphasic morphology with coexistence of squamous epithelial and sarcomatous spindle-cell components. Yellow, red, and green boxes indicate the sarcomatous component, squamous epithelial component, and areas of treatment-related hyalinization with inflammatory infiltrates, respectively. (**B**) High-power view of the squamous cell carcinoma component. (**C**) High-power view of the sarcomatous spindle-cell component. (**D**) Hyalinization with inflammatory infiltrates predominantly composed of lymphocytes and foamy histiocytes within the sarcomatous component, suggesting a chemotherapy-induced therapeutic effect. (**E**) Transitional area between squamous epithelial and sarcomatous spindle-cell components. (**F**) CK AE1/AE3 staining demonstrating positivity in the epithelial component and negativity in the sarcomatous component. (**G**) Vimentin staining predominantly observed in the sarcomatous component. CK, cytokeratin

A woman in her 50s presented with discomfort on swallowing. Her history included treated hepatitis C and a right breast fibroadenoma; she had smoked 4–5 cigarettes/day for only 1 year and drank alcohol occasionally. Tumor markers (SCC, CEA, CA19-9, CA125) were within normal limits. Upper gastrointestinal endoscopy revealed a type-1 polypoid tumor centered on the posterior wall 18 cm from the incisors, just distal to the esophageal inlet (17 cm) (**Fig. 1A** and **1B**). Because of the bulky protruding morphology, precise evaluation of superficial epithelial extension by iodine staining was limited at the initial examination. Hematoxylin–eosin staining of biopsy specimens showed a spindle-cell–predominant tumor, and IHC (cytokeratin AE1/AE3, p63) identified an epithelial component, leading to a diagnosis of carcinosarcoma with admixed epithelial and mesenchymal elements (**Fig. 2**). Contrast-enhanced CT scan demonstrated an approximately 6.5-cm irregular exophytic lesion in the cervical esophagus extending to the upper thoracic esophagus, without evidence of extramural invasion (**Fig. 1C**). PET-CT scan also revealed intense uptake at the same site (SUVmax 15.3) with no evidence of nodal or distant metastasis. The clinical stage was cT3N0M0, cStage II (UICC TNM 8th edition). In accordance with our institutional treatment strategy for locally advanced esophageal squamous cell carcinoma, and because no standard treatment has been established for esophageal carcinosarcoma, we selected neoadjuvant DCF therapy followed by curative surgery. In addition, because the patient strongly wished to preserve laryngeal function and there was no clear evidence of hypopharyngeal invasion, laryngeal preservation was considered a potential possibility. Therefore, treatment response was carefully evaluated during neoadjuvant therapy. After confirming a favorable response with marked tumor shrinkage following the initial course, a second cycle of DCF was administered (**Fig. 3A**–**3C**). Subsequent contrast-enhanced CT and endoscopy showed a marked response, with only a minimal ~5-mm proximal extension toward the right posterior wall remaining (**Fig. 3D**); we therefore judged curative resection with laryngeal preservation to be feasible. The patient underwent robot-assisted thoracoscopic subtotal esophagectomy with 3-field lymphadenectomy and posterior mediastinal reconstruction with cervical esophagogastric anastomosis using a 23-mm powered circular stapler (**Fig. 4A**–**4D**). During proximal esophageal transection, intraoperative iodine staining delineated the lesion boundary (**Fig. 4E**), enabling laryngeal preservation. Operative time was 9 h 54 min and blood loss was 70 mL. The postoperative course was uneventful, and the patient was discharged on POD 16. No adjuvant chemotherapy was given. Under strict surveillance, no recurrence or metastasis has been detected on imaging at 12 months postoperatively. On the resected specimen, a 3-cm tumor centered on the posterior wall was identified. Pathologic staging was pT1b-SM1 with no lymph node metastasis, and the treatment effect was grade 1a (**Fig. 5**). The proximal resection margin was negative. Histology showed a biphasic tumor with a continuous transition between atypical squamous epithelial cells and spindle-shaped mesenchymal cells, consistent with carcinosarcoma. The sarcomatous component exhibited inflammatory infiltrates predominantly composed of lymphocytes and foamy histiocytes, with focal hyalinization, suggesting a therapeutic effect of chemotherapy on the sarcomatous element as well (**Fig. 6**).

## DISCUSSION

Esophageal carcinosarcoma is a rare tumor composed of epithelial and mesenchymal elements, and the available evidence remains limited. Surgery is generally the mainstay of treatment, whereas the efficacy of chemotherapy and radiotherapy is inconsistent and no standard regimen has been established.^3)^ Although resected cases can achieve relatively favorable long-term outcomes, the role of neoadjuvant or adjuvant therapy in addition to surgery is considered limited.^3)^ With respect to neoadjuvant therapy, regimens based on cisplatin plus 5-fluorouracil and taxane-containing combinations have been reported; however, real-world cohort studies often conclude that efficacy is limited, leaving regimen selection to case-by-case judgment.^1)^ Conversely, several reports describe dramatic tumor shrinkage—including in the sarcomatoid component—and even pathological complete responses after neoadjuvant DCF, suggesting a potential advantage of taxane-containing therapy.^4)^ Taxane-based regimens have also demonstrated activity in certain soft tissue sarcomas, which may partly explain the observed response in the sarcomatous component in the present case. Taxane-containing regimens such as DCF have demonstrated superior response rates compared with conventional CF in advanced esophageal squamous cell carcinoma, as shown in the JCOG1109 study.^5)^ Regarding radiotherapy, while it has established roles in definitive and salvage settings for esophageal cancer as a whole,^6)^ data specific to carcinosarcoma are scarce; a single-center retrospective study of intensity-modulated radiation therapy (with literature review) suggested safety and potential efficacy only in a limited context.^7)^ Overall, broader generalization of systemic and radiation approaches will require further accumulation of well-documented cases.

In the present case of cervical esophageal carcinosarcoma, we selected neoadjuvant DCF as for conventional esophageal squamous cell carcinoma in accordance with our institutional policy (typically 2 courses followed by curative resection). After observing a favorable response to the first course, we administered a second, achieving marked tumor reduction and enabling laryngeal preservation. Although the pathological regression was graded as 1a, substantial tumor downsizing was achieved radiologically, which was sufficient to permit laryngeal preservation. Locally advanced cervical esophageal cancers often necessitate concurrent pharyngolaryngectomy; however, appropriate neoadjuvant therapy coupled with meticulous determination of resection boundaries can expand the indications for larynx-preserving surgery. Conversely, if sufficient tumor regression had not been achieved after neoadjuvant chemotherapy, additional treatment strategies such as chemoradiotherapy/radiotherapy or pharyngolaryngoesophagectomy would have been considered depending on resectability and expected surgical margins. In this patient, neoadjuvant DCF and rigorous assessment of the proximal margin allowed cervical esophagectomy with gastric conduit reconstruction under laryngeal preservation, helping to maintain voice and swallowing and thereby improving postoperative QOL. For intraoperative margin planning, we used Lugol’s iodine staining to visualize the lesion boundary and secure a safe proximal margin. Lugol’s iodine staining exploits the glycogen affinity of normal squamous epithelium and is a classical, useful method for assessing proximal margins in esophageal cancer. Pathologically, although substantial squamous cell carcinoma components were identified in the resected specimen, the initial biopsy specimen was predominantly composed of spindle-shaped atypical cells. Furthermore, the sarcomatous component exhibited inflammatory infiltrates predominantly composed of lymphocytes and foamy histiocytes, with focal hyalinization, suggesting that the therapeutic effect extended to the sarcomatous element as well. In addition, immunohistochemical staining demonstrated diffuse p53 overexpression in both the squamous epithelial and sarcomatous components, supporting a common clonal origin of the biphasic tumor (**Supplementary Fig. 1**).

Prognostically, favorable survival rates have been reported in resected series (e.g., 3-year OS; 83.3%, 5-year OS; 70.8%),^3)^ and several studies suggest a trend toward better survival than conventional squamous cell carcinoma.^8)^ In addition, macroscopic tumor morphology has been reported as a significant prognostic factor in esophageal spindle-cell carcinoma, a histological variant closely related to carcinosarcoma, with ulcerative-type tumors associated with poorer outcomes compared with polypoid-type tumors.^9)^ However, pathological nodal metastasis (pN) is a strong adverse factor.^2,10)^ The benefit of adjuvant therapy (chemotherapy and/or radiotherapy) remains unclear, and although adjuvant nivolumab prolonged disease-free survival in esophageal cancer overall,^11)^ direct application to carcinosarcoma is problematic. Individualized decision-making based on stage, pN status, margins, and general condition is therefore essential; for early-stage, node-negative, R0-resected cases without evidence of residual disease, careful observation may be reasonable. This case illustrates that a strategy combining neoadjuvant chemotherapy with function-preserving surgery can be effective even for cervical esophageal carcinosarcoma. Taken together, these findings suggest that taxane-containing neoadjuvant chemotherapy may be particularly beneficial in selected patients with cervical esophageal carcinosarcoma. Prospective accumulation of cases is warranted to refine regimen selection and to clarify the roles of radiotherapy and immunotherapy in this histology.

## CONCLUSIONS

This exceptionally rare case of cervical esophageal carcinosarcoma demonstrated a favorable response to neoadjuvant DCF, enabling curative resection with laryngeal preservation through tumor downsizing. The findings highlight potential chemosensitivity of the sarcomatous component and the clinical value of function-preserving surgery, underscoring the need for further case accumulation to establish optimal treatment strategies.

## SUPPLEMENTARY MATERIALS

Supplementary Figure 1 Immunohistochemical staining for p53 in esophageal carcinosarcoma. (**A**) Low-power view demonstrating diffuse nuclear overexpression of p53 in both the squamous epithelial component (right side) and sarcomatous spindle-cell component (left side). (**B**) High-power view of the sarcomatous spindle-cell component showing strong nuclear p53 positivity. (**C**) High-power view of the squamous epithelial component showing diffuse nuclear p53 overexpression. These findings support a common clonal origin of the biphasic tumor components.

## DECLARATIONS

### Availability of data and materials

The datasets supporting the conclusions of this article are included within the article.

## References

[1] Xu X, Xu Y, Wang J, et al. The controversy of esophageal carcinosarcoma: a case report and brief review of literature. Medicine (Baltimore) 2019; 98: e14787. 30855491 10.1097/MD.0000000000014787 PMC6417540

[2] Shen J, Lu K, Liu F, et al. Clinicopathologic features and surgical treatment prognosis of esophageal carcinosarcoma. Front Oncol 2024; 14: 1387611. 39234394 10.3389/fonc.2024.1387611 PMC11371597

[3] Chen S, Shi Y, Lu Z, et al. Esophageal carcinosarcoma: analysis of clinical features and prognosis of 24 cases and a literature review. Cancer Control 2021; 28: 10732748211004886. 33998308 10.1177/10732748211004886 PMC8204522

[4] Yoshimoto T, Kobayashi S, Kanetaka K, et al. Preoperative chemotherapy with docetaxel, cisplatin, and 5-fluorouracil for locally advanced esophageal carcinosarcoma: a case report and review of the literature. Surg Case Rep 2018; 4: 18. 29455418 10.1186/s40792-018-0425-4 PMC5816731

[5] Kato K, Machida R, Ito Y, et al. Doublet chemotherapy, triplet chemotherapy, or doublet chemotherapy combined with radiotherapy as neoadjuvant treatment for locally advanced oesophageal cancer (JCOG1109 NExT): a randomised, controlled, open-label, phase 3 trial. Lancet 2024; 404: 55–66. 38876133 10.1016/S0140-6736(24)00745-1

[6] Ogasawara N, Tamura Y, Funaki Y, et al. Rapidly growing esophageal carcinosarcoma reduced by neoadjuvant radiotherapy alone. Case Rep Gastroenterol 2014; 8: 227–34. 25076867 10.1159/000365320 PMC4105955

[7] Yang S, Wang W, Bi N, et al. Intensity modulated radiotherapy might be effective for locally advanced esophageal carcinosarcoma: a single center’s experience and review of literature. Medicine (Baltimore) 2022; 101: e31215. 36281080 10.1097/MD.0000000000031215 PMC9592314

[8] Hu B, Zhao K, Yang Y, et al. Investigating esophageal sarcomatoid carcinoma and its comparison with esophageal squamous cell carcinoma on clinicopathological characteristics, prognosis, and radiomics features: a retrospective study. Front Oncol 2024; 14: 1398982. 39011471 10.3389/fonc.2024.1398982 PMC11247005

[9] Hashimoto M, Kitagami H, Niwa H, et al. Prognosis and prognostic factors of esophageal spindle cell carcinoma treated by esophagectomy: a retrospective single-institution analysis. Esophagus 2019; 16: 292–9. 30937574 10.1007/s10388-019-00667-y

[10] Iyomasa S, Kato H, Tachimori Y, et al. Carcinosarcoma of the esophagus: a twenty-case study. Jpn J Clin Oncol 1990; 20: 99–106. 2319703

[11] Kelly RJ, Ajani JA, Kuzdzal J, et al. Adjuvant nivolumab in resected esophageal or gastroesophageal junction cancer. N Engl J Med 2021; 384: 1191–203. 33789008 10.1056/NEJMoa2032125

