# Supplementary figures and images for: Neoadjuvant Docetaxel/Cisplatin/5-Fluorouracil Enabling Laryngeal Preservation in Cervical Esophageal Carcinosarcoma: A Case Report
Source: Surg Case Rep. 2026 Jul 7;12(1):26-0277. doi: 10.70352/scrj.cr.26-0277 (PMC13345686; doi:10.70352/scrj.cr.26-0277)

Supplementary Fig. S1.

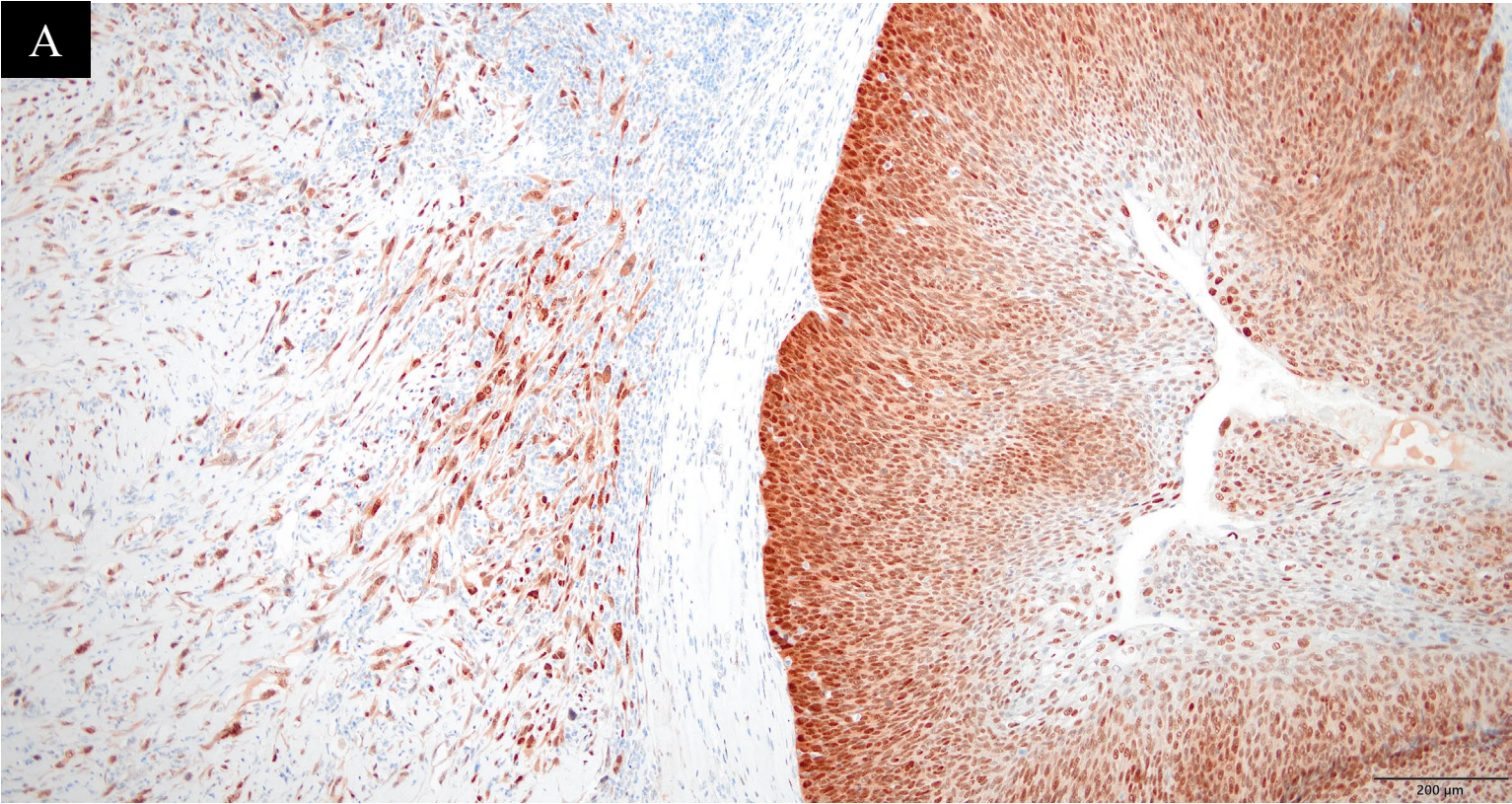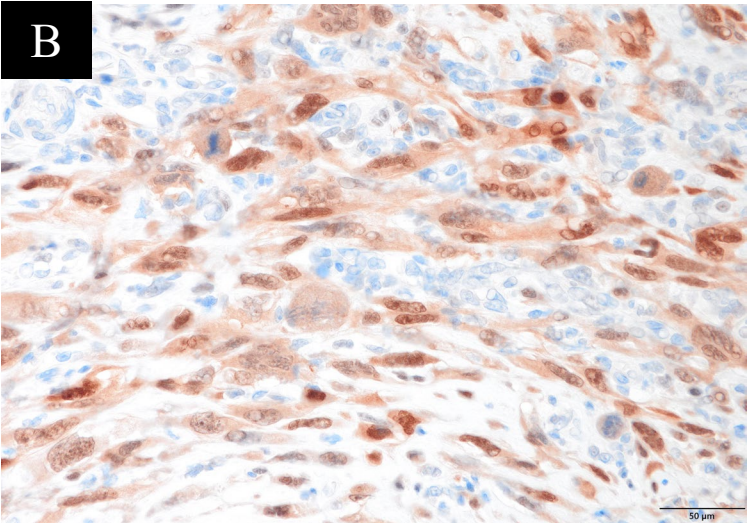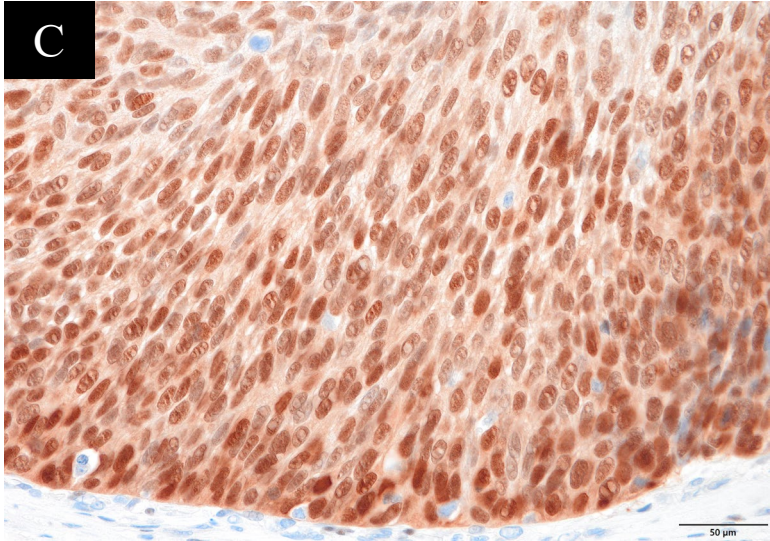

Supplement: Supplementary Figure 1 — Immunohistochemical staining for p53 in esophageal carcinosarcoma. (A) Low-power view demonstrating diffuse nuclear overexpression of p53 in both the squamous epithelial component (right side) and sarcomatous spindle-cell component (left side). (B) High-power view of the sarcomatous spindle-cell component showing strong nuclear p53 positivity. (C) High-power view of the squamous epithelial component showing diffuse nuclear p53 overexpression. These findings support a common clonal origin of the biphasic tumor components. [file scr-12-01-26-0277-s001.pdf]
